# Genetic Tendency Analysis and Comprehensive Antioxidant Activity Evaluation of Leaves and Flowers of Loquat F_1_ Generation

**DOI:** 10.3390/cimb47010058

**Published:** 2025-01-16

**Authors:** Qixuan Zhu, Xiaoying Li, Hang Ge, Zhixuan Wang, Binjun Wang, Junwei Chen, Hongxia Xu

**Affiliations:** 1 Institute of Horticulture, Zhejiang Academy of Agricultural Sciences, Hangzhou 310021, China; zhu_qixuan@163.com (Q.Z.); muzixiaohaitun@126.com (X.L.); geh@zaas.ac.cn (H.G.); wzx107785123@163.com (Z.W.); 17857323821@139.com (B.W.); chenjunwei@zaas.ac.cn (J.C.); 2 College of Horticulture Science & Technology, Hebei Normal University of Science & Technology, Qinhuangdao 066600, China

**Keywords:** loquat, total phenolics, flavonoids, antioxidant activity, correlation

## Abstract

Loquat leaves, flowers, and other organs contain abundant antioxidant substances, which have wide applications in medicine, health, and food industries. This study aims to provide theoretical guidance for loquat hybrid parent and combination selection and a basis for high-quality loquat strain screening and development. For comprehensive antioxidant profiling, we used “Ninghaibai” and “Oobusa” loquat and their F_1_ generation as experimental materials to determine the total phenol, flavonoid, DPPH, ABTS, and FRAP content in the leaves and flowers of 56 strains. Five traits, including total phenols, flavonoids, DPPH, ABTS, and FRAP, were widely separated and normally distributed in the flowers of 56 F_1_ loquat strains, exhibiting the genetic basis of these quantitative traits. However, these traits displayed widely separated and slightly skewed distribution in the leaves of the F_1_ generation. The total phenols, flavonoids, DPPH, and FRAP showed a trend of small inheritance in the leaves. However, the ABTS showed a trend of medium and high inheritance in leaves and flowers, respectively. Through cluster and principal component analyses, a comprehensive antioxidant activity evaluation was conducted. Ten strains with comprehensive scores greater than 1 for antioxidant activity in leaves and flowers were selected. Among them, the top three strains with high antioxidant capacity were ND107, “Oobusa”, and ND128. These results suggest that hybrid breeding guided by the genetic characteristics of each trait can improve the possibility of cultivating new varieties with high antioxidant activity.

## 1. Introduction

Loquat (*Eriobotrya japonica* L.) is a perennial evergreen fruit tree of Rosaceae family, which is native to China and widely cultivated worldwide. The entire plant, encompassing its fruits, leaves, flowers, seeds, and bark, has been utilized and sold for medicinal purposes since ancient times. Loquat leaves and flowers possess high concentrations of phenolics and triterpenic acid, whereas the fruit is rich in phenolics, carotenoids, sugars, organic acids, and various vitamins [1]. These compounds exhibit diverse biological effects, such as antiviral, anti-inflammatory, antiasthmatic, antidiabetic, and anticancer activity [2]. Furthermore, loquat possesses notable antioxidant and free-radical-scavenging properties, which are closely associated with polyphenolic compounds, phenolic acids, and flavonoids [3,4,5,6]. Zhou et al. [6] conducted an antioxidant analysis on loquat flower extract and found that the ABTS method had the highest correlation between antioxidant activity and total phenolic and flavonoid content. Xu et al. [7] demonstrated that flavonoids and total phenols are the main antioxidant components in loquat fruit. Zhou et al. [8] examined the phenolic content and antioxidant activity in loquat fruit from 24 different cultivars grown in China and observed that phenolic compounds contribute significantly to hydrophilic antioxidant activity, whereas carotenoids play a significant role in lipophilic antioxidant activity.

Phenolic compound accumulation is significantly affected by tissues, developmental stage, growth environment, and genetic background [9,10,11,12]. Zhou et al. [6] showed that the highest flavonoid concentration was observed in full-bloom-stage flowers, with petals containing the highest phenolic concentration. Xu et al. [10] analyzed the fruit quality of 12 loquat varieties and found significant differences in total phenolic and flavonoid content as well as antioxidant activity among different varieties. Hong et al. [8] found that the phenolic content and antioxidant activity in wild loquat leaves were higher than those in cultivated loquat leaves. Moreover, there were differences in total phenolic and flavonoid content and antioxidant activity among different organs.

Hybrid breeding is one of the important means to improve the genetic traits of fruit trees. Understanding heritability and heterosis is crucial for crop improvement. Ning et al. [13] found significant differences in total phenol and flavonoid content and antioxidant activity among different strains of hybrid offspring. Kaczmarska et al. [14] found that the chemical composition variation among strawberry fruits across different genotypes was significantly influenced by the specific pairings of individual genotypes, and certain combinations exhibited heterosis in phytochemical content. Karaat and Serce [15] highlighted the substantial impact of genotypic factors on the expression of fruit quality characteristics within a given offspring group. Their study revealed high heritability for most pomological traits, while total phenolic content and TEAC exhibited moderate to low levels of inheritance in apricot progenies. Mihaylova et al. [16] found that the interspecific hybrids of plum and apricot exhibited higher total phenolic, flavonoid, and antioxidant activities compared to their parental varieties. Recently, Kumar et al. [17] published a review that reports heterosis in *Solanum melongena* traits, such as yield, plant height, fruit weight, ascorbic acid content, total phenolic content, and early maturity. Butcher et al. [18] found positive heterosis for capsaicin and total capsaicinoid and negative heterosis for quercetin and luteolin in *Capsicum annuum*. Baipai et al. [19] determined the heterotic patterns of primary and secondary metabolites in the oilseed crop *Brassica juncea*, and the tested metabolites displayed both additive and non-additive modes of inheritance in F_1_ hybrids.

Loquat leaves, flowers, and other organs contain abundant antioxidant substances, which have been widely used in the medicine, health, and food industries. Most of the research on phenolic compounds and antioxidant activity in loquat only focuses on different secondary metabolite content between tissues or species, while reports on the genetic predisposition analysis of loquat antioxidant activity and the breeding of high antioxidant varieties are very limited. This work determined total phenolic and flavonoid content and antioxidant activity in the leaves and flowers of the segregating F_1_ population derived from a cross between “Ninghaibai” × “Oobusa”. The objective of the study was to explore the diversity and genetic tendency of active compounds and antioxidant activity in the leaves and flowers of loquat hybrid offspring. The findings contribute to the understanding of the inheritance pattern of functional traits and are conducive for the breeding success of loquat with superior active compounds.

## 2. Materials and Methods

### 2.1. Plant Materials

Fifty-six F_1_ plants and their parents from the hybrid population of “Ninghaibai” and “Oobusa” planted at the Haining Yangdu Innovation Base of Zhejiang Academy of Agricultural Sciences were used as experimental materials. In September 2023, 20 disease-free spring shoot leaves were collected from the middle and upper parts of the outer crown of each individual tree, washed with clean water, dried, and veins removed and cut into pieces. After being treated with liquid nitrogen, these samples were stored at −80 °C. From November to December 2023, 20 flower spikes were collected from the middle and upper parts of the outer crown of each individual tree. The buds in the flower spikes were taken, treated with liquid nitrogen, and stored in an ultra-low-temperature refrigerator at −80 °C for further use. A genetic tendency analysis and comprehensive evaluation of the antioxidant components and activities of the collected leaf and flower samples were carried out.

### 2.2. Methods and Materials

#### 2.2.1. Materials

Methanol (A506806), gallic acid (A600476), Folin–Ciocalteu (A500467), sodium carbonate (A424705), rutin (A610616), sodium nitrite (A415326), aluminum chloride hexahydrate (A500025), sodium hydroxide (A620617), Trolox (6-hydroxy-2,5,7,8-tetramethylchroman-2-carboxylic acid) (A426709), DPPH (the 1,1-diphenyl-2- picrylhydrazyl radical) (A429496), ABTS [2,2-azino-bis (3-ethylbenzthiozoline-6-sulfonic acid)] (A600002), potassium persulfate (A100363), ethanol absolute (A500737), iron(II) sulfate heptahydrate (A600461), sodium acetate trihydrate (A110530), acetic acid (A501931), TPTZ [2,4,6-tri(2-pyridyl)-s-triazine] (A413858), and iron(III) chloride hexahydrate (A600201) were all purchased from Sangon Biotech (Shanghai, China). All reagents were of analytical grade unless indicated otherwise.

#### 2.2.2. Instruments

An ultra-low-temperature refrigerator (DW-86L388J, Haier, Qingdao, China), high-throughput tissue grinder (Ck2000, Thmorgan, Beijing, China), centrifuge (5804 R, Eppendorf, Hamburg, Germany), and ELISA reader (Gen5, BioTek, Winooski, VT, USA) were utilized.

#### 2.2.3. Extracts for Phenolic and Antioxidant Capacity Measurement

The preparation of the extract and determination of antioxidant components and activities were carried out according to the method of Xu et al. [10]. Loquat leaf and flower samples were ground into powder using a high-throughput tissue grinder. A powdered sample (0.5 g) was accurately weighed and 25 mL of anhydrous methanol was added and stirred evenly. The sample was kept at 4 °C for 12 h, and centrifuged at 10,000× *g* for 20 min. The supernatant was collected and stored at −20 °C for the determination of total phenolic and flavonoid content and DPPH free radical scavenging, ABTS cation free radical scavenging, and iron ion reducing abilities. Each treatment was repeated three times, and the determination instrument was an ELISA reader.

#### 2.2.4. Total Phenolic Content Analysis

The total phenolic content was determined by the Folin–Ciocalteu colorimetric method. Gallic acid standard solutions (0, 0.1, 0.2, 0.4, 0.6, and 0.8 mg·mL^−1^) were prepared. The gradient concentration standard or test solutions (0.1 mL) were pipetted, which were diluted to 1 mL. Then, 5 mL of 0.2 mol·L^−1^ Folin–Ciocalteu reagent was added, mixed well, and allowed to stand for 5 min. Thereafter, 4 mL of 150 g·L^−1^ Na_2_CO_3_ solution was added and mixed well. The reaction solution (200 µL) was then pipetted onto an ELISA plate. Anhydrous methanol was used as a blank. The absorbance was read at 765 nm to calculate the total phenolic content. The experiment was repeated three times. The total phenolic content of the samples was estimated by the standard curve of gallic acid. The results were expressed as mg gallic acid equivalent (GAE)·g^−1^ fresh weight (FW).

#### 2.2.5. Total Flavonoid Content Analysis

The sodium nitrite–aluminum chloride method was used to determine the flavonoid content. Rutin standard solutions (0, 0.1, 0.2, 0.4, 0.6, and 0.8 mg·mL^−1^) were prepared. The gradient concentration standard or test solution (2 mL) was drawn and distilled water was added to make the sample up to 5 mL. NaNO_2_ (0.3 mL of 0.72 mol·L^−1^) was then added, mixed well, and allowed to stand for 5 min. Thereafter, AlCl_3_ (0.6 mL of 0.41 mol·L^−1^) was added, mixed well, and allowed to stand for 6 min. Next, 2 mL of 1 mol·L^−1^ NaOH and 2.1 mL of distilled water were added and mixed. The reaction solution (200 μL) was drawn onto the ELISA plate. Anhydrous methanol was used as a blank. The absorbance was read at 510 nm to calculate the flavonoid content. The experiment was repeated three times. The flavonoid content of the sample was estimated by the standard curve of rutin. The results were expressed as mg rutin equivalent (RE)·g^−1^ FW.

#### 2.2.6. Antioxidant Capacity Determination

##### Free Radical Scavenging Activity Using DPPH Assay

The test solution (0.1 mL) was pipetted, and 3 mL of 0.1 mol·L^−1^ DPPH methanol solution was added, mixed well, and allowed to react for 30 min. Then, 200 µL of the reaction solution was pipetted onto the ELISA plate. Anhydrous methanol was used as a blank. The absorbance was read at 517 nm to calculate the antioxidant activity. The experiment was repeated three times. The free radical scavenging activity of the sample was estimated by the standard curve of Trolox. The results were expressed as µmol Trolox equivalent (TE)·g^−1^ FW.

##### Antioxidant Activity Using ABTS Assay

The test solution (0.2 mL) was pipetted, 2 mL of 7 mmol·L^−1^ ABTS reaction solution was added, 2.45 mmol·L^−1^ K_2_S_2_O_8_ solution was allowed to react in the dark for 16 h, diluted with ethanol to an absorbance of 0.700 at 734 nm, mixed well, and allowed to react for 15 min. Then, 200 µL of the reaction solution was pipetted onto an ELISA plate. Anhydrous methanol was used as a blank. The absorbance was read at 734 nm to calculate the antioxidant activity. The experiment was repeated three times. The antioxidant activity of the sample was estimated by the standard curve of Trolox. The results were expressed as µmol TE·g^−1^ FW.

##### Ferric-Reducing/Antioxidant Power Assay

The FRAP reaction solution was prepared as follows. Acetate buffer (25 mL of 300 mmol·L^−1^; 3.1 g C_2_H_3_NaO_2_·3H_2_O, 16 mL C_2_H_4_O_2_, pH 3.6), 2.5 mL of 10 mmol·L^−1^ TPTZ solution (40 mmol·L^−1^ HCl), and 2.5 mL of 20 mmol·L^−1^ FeCl_3_·6H_2_O solution were mixed to prepare a fresh reaction solution, which was heated at 37 °C prior to use. The test solution (0.15 mL) was pipetted, and 2.85 mL of FRAP reaction solution was added, mixed, and placed in water bath at 37 °C in the dark for 30 min. The reaction solution (200 µL) was pipetted onto an ELISA plate. Anhydrous methanol was used as a blank. The absorbance was read at 593 nm to calculate the antioxidant activity. The experiment was repeated three times. The ferric-reducing/antioxidant power of the sample was estimated by the standard curve of iron(II) sulfate. The results were expressed as µmol iron(II) sulfate equivalent (ISE)·g^−1^ FW.

### 2.3. Statistical Analysis

The data were processed using Excel 2016. Correlation heatmaps, biplots, and cluster heatmaps were drawn using Origin 2021, and principal component analysis was performed using IBM SPSS Statistics 26.0. The calculation formulae for genetic parameters were as follows: coefficient of variation (*CV*, %) = (S/F) × 100; genetic transmission ability (*Ta*, %) = (F/MP) × 100; mid-parent heterosis rate (MPH, %) = [(F-MP)/0.5 (P1 + P2)] × 100; over-high parent heterosis rate (HH, %) = ∑hp/n × 100; inferior-low parent heterosis rate (LH, %) = ∑lp/n × 100. In these formulae, S indicates the standard deviation; F represents the average value of F_1_ generation; MP signifies the middle parent value; P1 and P2 represent the parent value; hp symbolizes the number of F_1_ plants with phenotypic values higher than the high parent; lp denotes the number of F_1_ plants with phenotypic values lower than the low parent; and n represents the total number of populations.

## 3. Results

### 3.1. Genetic Analysis of Antioxidant Components and Activities of F_1_ Leaves of “Ninghaibai” and “Oobusa”

**Figure 1:**
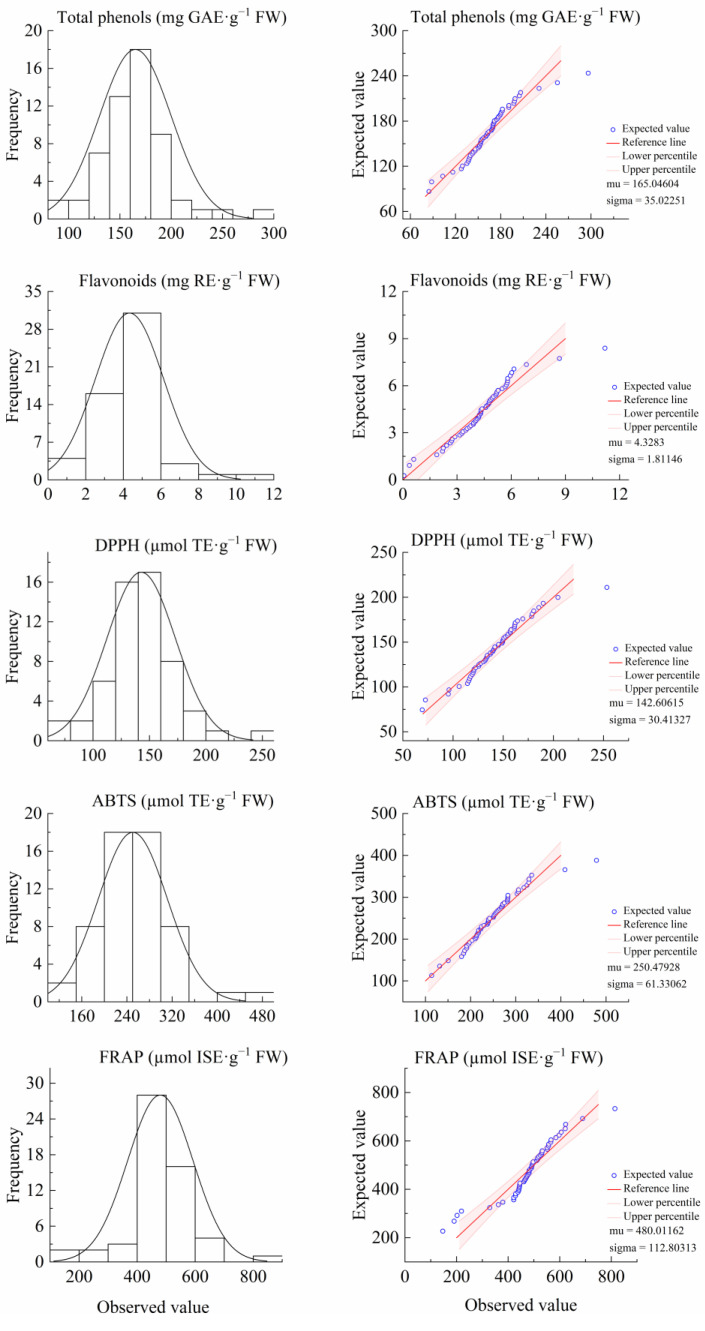
Histogram (**left**) and normal Q-Q plot (**right**) of antioxidant components and activity of F_1_-generation leaves from loquats “Ninghaibai” and “Oobusa”.

As shown in Figure 1 and Table 1, in the F_1_ leaves, the total phenols, flavonoids, DPPH, ABTS, and FRAP were continuously distributed, and the kurtosis and skewness were consistent with skewed distribution characteristics. The results of the genetic analysis of F_1_-generation leaf traits are shown in Table 1. The average values of total phenols, flavonoids, DPPH, and FRAP in the F_1_ leaves were lower than those of the “Ninghaibai” (low parent value). The average value of ABTS in the F_1_ leaves was higher than that of “Ninghaibai”, but lower than that of the average value (middle parent value) of “Ninghaibai” and “Oobusa”. The range of separation for the five traits was extensive, with a significant disparity between the maximum and minimum values, particularly for flavonoids, where the maximum value was 186.5 times greater than the minimum value. The coefficient of variation was between 21.22% (total phenol content) and 41.85% (flavonoid content), and a high degree of trait separation was observable. There was rich genetic diversity; the genetic transmission ability was between 54.80% (flavonoids) and 83.40% (ABTS), the mid-parent heterosis rate was between −45.20% (flavonoids) and −16.60% (ABTS), and the genetic transmission ability and mid-parent heterosis rate of the traits were low. In the F_1_-generation leaves, the total phenols, flavonoids, DPPH, and FRAP showed a trend of small inheritance, whereas ABTS showed a trend of medium inheritance. It may be that the non-additive effects of the parents were strong and became disintegrated during hybridization, resulting in trait decline. However, strains with strong antioxidant activity still appeared in the hybrid offspring, which were worth further breeding.

### 3.2. Genetic Analysis of Antioxidant Components and Activities of F_1_ Flowers of “Ninghaibai” and “Oobusa”

**Figure 2:**
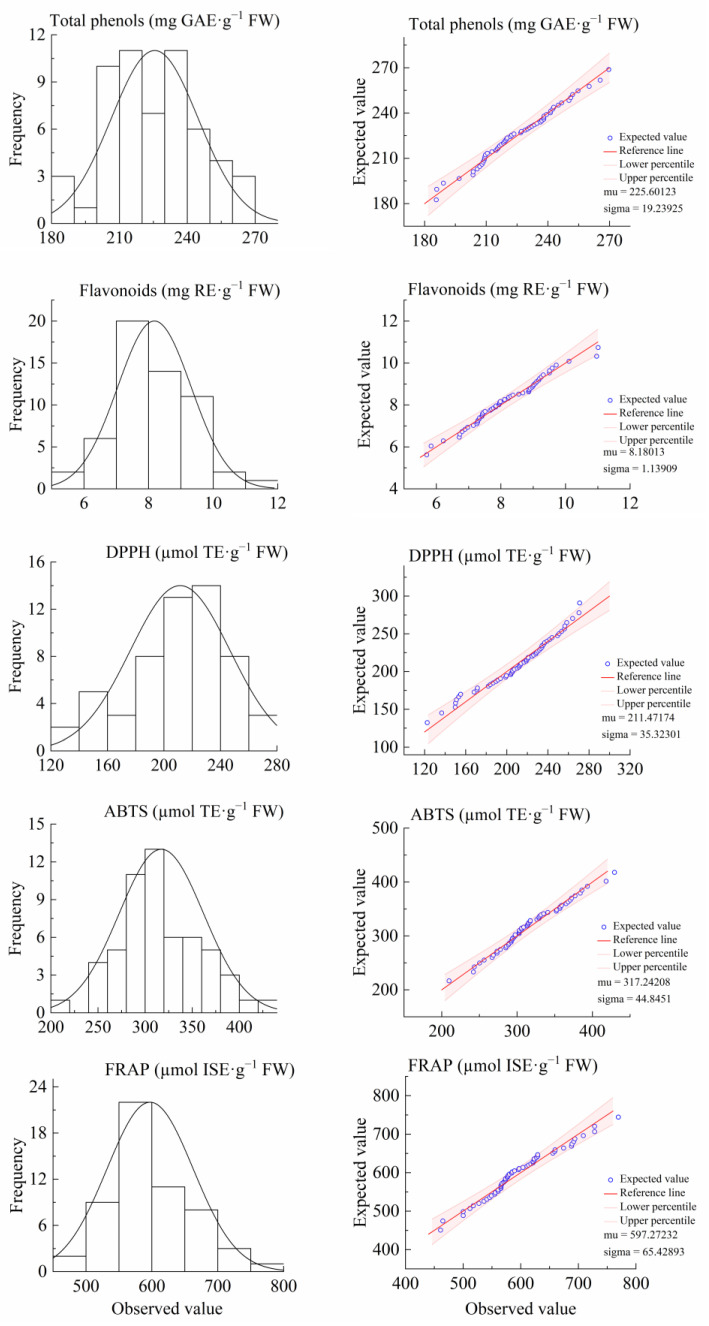
Histogram (**left**) and normal Q-Q plot (**right**) of antioxidant components and activity of F_1_-generation flowers from loquats “Ninghaibai” and “Oobusa”.

As shown in Figure 2 and Table 2, in the F_1_-generation flowers, the total phenols, flavonoids, DPPH, ABTS, and FRAP were continuously distributed, with small kurtosis and skewness, which was consistent with normal distribution characteristics. These were quantitative traits controlled by multiple genes; thus, further hybrid genetic analysis could be performed. The results of the genetic analysis of the F_1_-generation flower traits are shown in Table 2. The average values of total phenols and ABTS in F_1_ flowers were higher than those of the high parent value, and the average values of flavonoids, DPPH, and FRAP in the F_1_ flowers were lower than those of the low parent value. The coefficient of variation was between 8.53% (total phenols) and 16.70% (DPPH). Except for total phenols, the other traits showed a certain separation phenomenon in the F_1_ generation; the genetic transmission ability was between 91.68% (FRAP) and 105.79% (ABTS), and the mid-parent heterosis rate was between −8.32% (FRAP) and 5.79% (ABTS). In the F_1_-generation flowers, the total phenols, flavonoids, DPPH, and FRAP showed a trend of small inheritance, whereas ABTS showed a trend of high inheritance. In addition, the over-high parent heterosis rate of total phenols, flavonoids, DPPH, and FRAP in the F_1_-generation flowers were 48.21%, 35.71%, 41.07%, and 16.07%, respectively, thus meeting the demand for materials for breeding loquat varieties with stronger antioxidant activity in their flowers.

### 3.3. Comprehensive Evaluation Based on Antioxidant Activity and Components of F_1_ Leaves and Flowers

#### 3.3.1. Correlation Analysis of Antioxidant Components and Activity of F_1_ Leaves and Flowers

**Figure 3:**
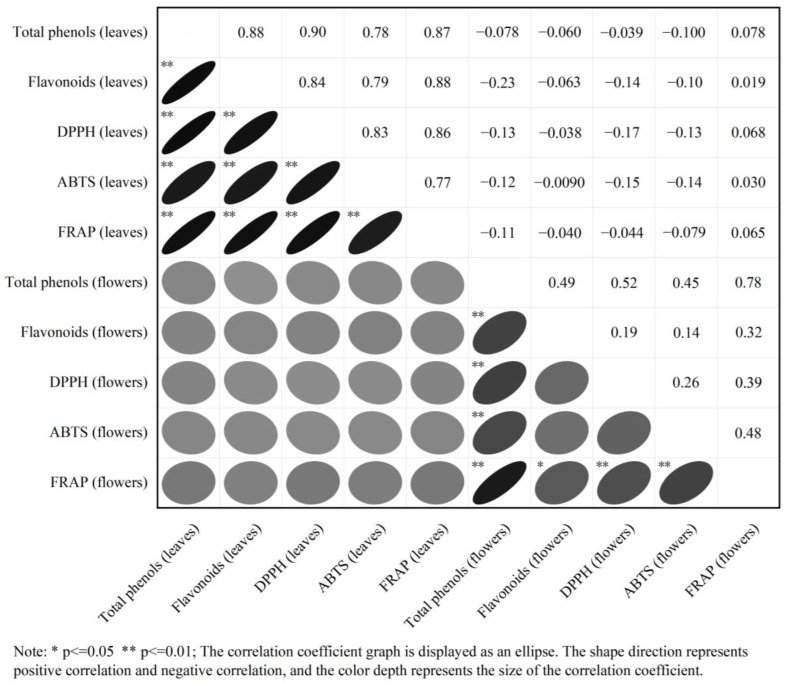
Pearson correlation analysis of antioxidant components and activity in F_1_-generation plants and their parent leaves and flowers from loquats “Ninghaibai” and “Oobusa”. Note: * *p* ≤ 0.05 ** *p* ≤ 0.01.

As shown in Figure 3, in the F_1_-generation leaves, a highly significant positive correlation was observed between total phenols and flavonoids, DPPH, ABTS, and FRAP, with correlation coefficients of 0.88, 0.90, 0.78, and 0.87, respectively. Further, a highly significant positive correlation was noticed between flavonoids and total phenols, DPPH, ABTS, and FRAP, with correlation coefficients of 0.88, 0.84, 0.79, and 0.88, respectively. In the F_1_ flowers, a highly significant positive correlation existed between total phenols and flavonoids, DPPH, ABTS, and FRAP, with correlation coefficients of 0.49, 0.52, 0.45, and 0.78, respectively. Further, a highly significant positive correlation was observed between flavonoids and total phenols, with a correlation coefficient of 0.49, and a significant positive correlation was noted between flavonoids and FRAP, with a correlation coefficient of 0.32. However, flavonoids were insignificantly correlated with DPPH and ABTS. Based on the above speculation, total phenols and flavonoids may be important functional components for the antioxidant activity of loquat leaves and flowers. In addition, there was no obvious correlation between antioxidant components and activity in the loquat leaves and flowers.

#### 3.3.2. Principal Component and Cluster Analyses of Comprehensive Antioxidant Activity of F_1_-Generation Leaves and Flowers

**Figure 4:**
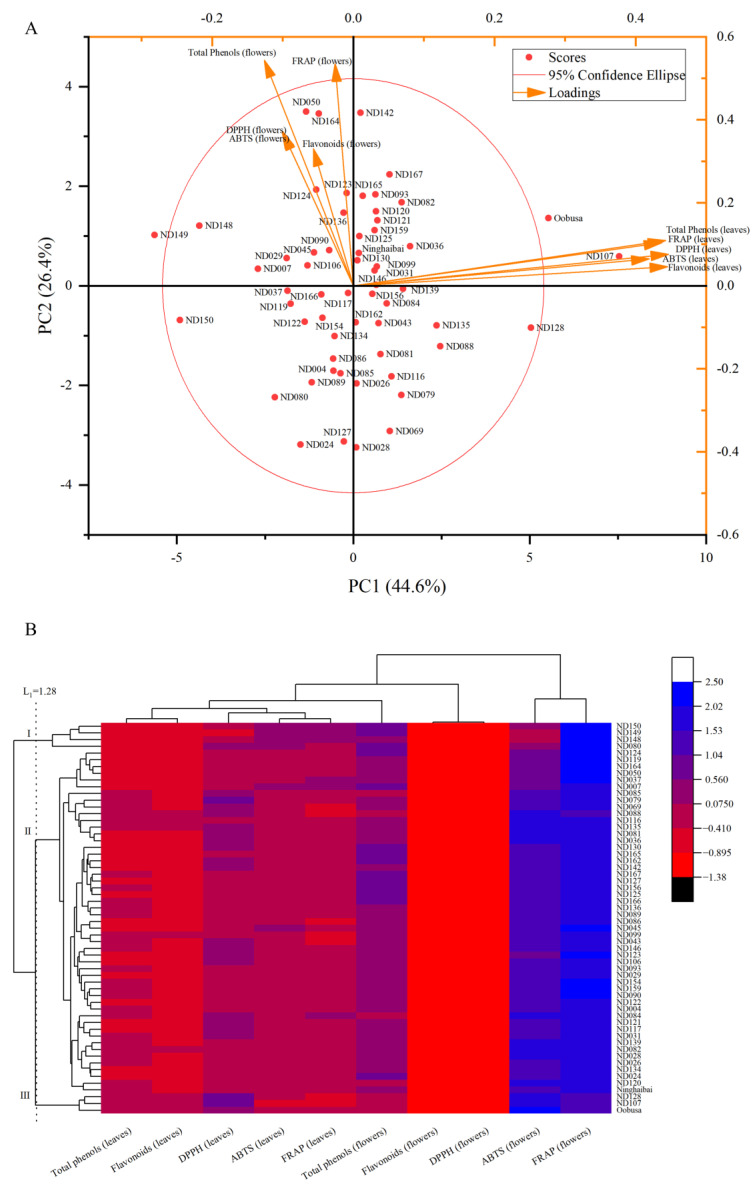
Principal component and cluster analyses of antioxidant components and activity in F_1_-generation plants and their parent leaves and flowers of loquat “Ninghaibai” and “Oobusa”. Note: (**A**): principal component analysis; (**B**): cluster analysis.

As shown in Figure 4A, among the 56 hybrid offspring and their parents, except for ND149, “Oobusa”, and ND107, the remaining strains were within the same confidence ellipse, indicating that there were large differences in the comprehensive antioxidant capacity of leaves and flowers between the above three strains and the remaining strains. As shown in Figure 4B and Table 3, when the distance is 1.28, the 58 strains can be divided into three categories. Class I includes ND150, ND149, ND148, and ND080 with weak leaf antioxidant activity, Class II includes 51 strains with medium average values of each trait, and Class III includes ND128, ND107, and “Oobusa” with strong leaf antioxidant activity.

#### 3.3.3. Principal Component and Cluster Analyses of Comprehensive Antioxidant Activity of F_1_-Generation Leaves and Flowers 

The principal component analysis of comprehensive antioxidant activity is shown in Table 4. According to the principal component analysis, two principal components with eigenvalues greater than 1 were obtained, with a cumulative contribution rate of 70.973%, which basically covered the main information of the comprehensive antioxidant activity of the F_1_ leaves and flowers. Among them, the contribution rate of PC1 was 44.575%, which mainly reflected the antioxidant activity—total phenols, flavonoids, DPPH, ABTS, and FRAP—in the leaves; the contribution rate of PC2 was 26.397%, which mainly reflected the antioxidant activity—total phenols, flavonoids, DPPH, ABTS, and FRAP—in the flowers.

Two principal components were used to evaluate the comprehensive antioxidant activity of the leaves and flowers of “Ninghaibai” and “Oobusa” F_1_ generations. According to the standardized data, the component matrix, eigenvalue, and contribution rate were obtained using SPSS 26.0 software, and the principal component eigenvector coefficient C = principal component load/(corresponding to the principal component eigenvalue 1/2) was calculated. According to the coefficients of the principal component eigenvectors and values of 10 indicators normalized by the analysis software (X_1_~X_10_), the score function expressions of the two principal components were constructed as PC1 = 0.442X_1_ + 0.445X_2_ + 0.447X_3_ + 0.419X_4_ + 0.437X_5_ − 0.126X_6_ − 0.057X_7_ − 0.101X_8_ − 0.099X_9_ − 0.026X_10_ and PC2 = 0.109X_1_ + 0.046X_2_ + 0.076X_3_ + 0.065X_4_ + 0.103X_5_ + 0.543X_6_ + 0.331X_7_ + 0.373X_8_ + 0.369X_9_ + 0.534X_10_.

According to the relative variance contribution rate of the two principal components, the comprehensive evaluation model for the antioxidant activity of the F_1_ leaves and flowers was constructed: comprehensive score (PC) = 0.446PC1 + 0.264PC2. The model was used to calculate the comprehensive scores of the antioxidant activity of the leaves and flowers of each strain and rank them. The higher the score, the better the comprehensive antioxidant activity.

According to the scores and rankings of the 56 F_1_-generation strains and their parents (Table 5), the comprehensive scores of the antioxidant activity of the leaves and flowers of 10 strains, including ND107, “Oobusa”, ND128, ND082, ND167, ND142, ND036, ND135, and ND088 ranked 1 to 10, were greater than 1, and the comprehensive antioxidant activity was excellent. These strains can be used as key research objects for the breeding and genetic improvement of new loquat varieties with high antioxidant activity. The comprehensive scores of the 21 strains ranked 11 to 31 were between 0 and 1, and their comprehensive antioxidant activity was average. The comprehensive scores of the 27 strains ranked 32 to 58 were less than 0, and their comprehensive antioxidant activity was poor.

## 4. Discussion

Polyphenolic compounds are widely available in plants [20], and antioxidant activity is the most important property of phenolic substances. Parameters such as DPPH, ABTS, and FRAP can effectively evaluate primary antioxidant capacity [21]. Reports have shown a positive correlation between phenolic content and antioxidant activity in various plant species [22]. In addition, phenolics in plants can donate hydrogen or electrons and form stable free radical intermediates [23] and are, therefore, known as effective in vitro antioxidants, which play a very important role in human health and disease prevention [5].

There are significant differences in the phenolic content and antioxidant activity of loquat leaves and flowers among different varieties [6,8]. Hybrid breeding can take advantage of hybrid vigor, with the phenotypic advantage of F_1_-generation traits being better than those of the parents [24]. New varieties with excellent traits and in line with breeding objectives are selected from the hybrid offspring. Tohge et al. [25] reported the framework of tomato phenolic substance synthesis, indicating that phenolic substance synthesis is jointly participated in by many genes. In this study, the total phenols, flavonoids, DPPH, ABTS, and FRAP were found to be normally distributed in the flowers as quantitative traits controlled by polygenes, whereas they presented a slightly skewed distribution in the leaves due to the small sample size. In addition, there was a significant trait separation in the F_1_-generation leaves, with the coefficient of variation ranging from 21.22% (total phenols) to 41.85% (flavonoids). The trait separation was insignificant in the F_1_-generation flowers, and the variation coefficient ranged from 8.53% (total phenols) to 16.70% (DPPH). Muralidhara et al. [26] demonstrated the existence of extensive genetic variability in the F1 hybrid population of mango with regard to total phenols, flavonoids, and total antioxidants, emphasizing that genotypic variation holds dominance. Farhana et al. [27] showed large differences in total phenols and DPPH content among pepper F_1_ lines. In this study, the F1 generation leaves exhibited a greater variety of antioxidant components and activity than flowers, indicating superior genetic potential. This may be attributed to the close correlation of various traits in the parent flowers. Hence, when employing hybrid methods for breeding new varieties, it is imperative to select parents with significant differences for hybridization.

Genetic transmission ability can better reflect the extent to which traits are controlled by genetics [28], and the mid-parent heterosis rate can intuitively reflect the strength of hybrid vigor [29]. In this study, the antioxidant components and activities in flowers were less affected by the environment than in leaves, and they had certain hybrid vigor. Kaushik [30] reported that total phenols have significant hybrid vigor in eggplant. Bajpai et al. [19] reported that the heterosis of F_1_-generation phenolics in rapeseed differed in different tissues, with negative and mixed mid-parent dominance in leaves and buds, respectively. Total flavonoids and luteolin have positive hybrid vigor, whereas chlorogenic acid and isochlorogenic acid A have negative hybrid vigor. The genetic background of fruit trees is complex. During cross-breeding, the non-additive effects of genes are usually conducive for the phenotypic advantages of hybrid offspring, but there may be certain negative effects [31]. In addition, the high heterozygosity of parental genes is prone to the disintegration of non-additive effects during hybridization, and the offspring appear to have trait segregation. Therefore, the mutation rate of cross-breeding is generally high, but it is still possible to breed a certain number of super-high-parent plants into new varieties. Rapisarda et al. [32] reported that they utilized the hybrid vigor of citrus to develop offspring with significantly higher total phenols, total flavonoids, and antioxidant activities compared to their parental varieties. Ning et al. [13] reported that the super-parental separation of phenolic compounds occurred in the hybrid offspring of *Camellia chrysanthemum*. Among them, total flavonoids, luteolin, isochlorogenic acid A, and other traits showed a trend of high inheritance, whereas chlorogenic acid showed a trend of small inheritance. In this study, total phenols, flavonoids, DPPH, and FRAP showed a trend of small inheritance in the F_1_-generation leaves and flowers, whereas ABTS showed a trend of medium and high inheritance in the F_1_-generation leaves and flowers, respectively. The decline in the antioxidant composition and activity of F_1_-generation leaves and flowers was obvious, but offspring with higher antioxidant composition and activity still appeared in the F_1_-generation leaves. In the flowers, except for ABTS, the over-high parent heterosis rate of other traits was between 16.07 (FRAP) and 48.21 (total phenols), indicating that sufficient germplasm material can be provided for cultivating loquat varieties with high antioxidant activity.

In this study, a correlation analysis was conducted on the total phenol and flavonoid content and antioxidant activity in the leaves and flowers of the hybrid offspring of “Ninghaibai” and “Oobusa” loquats. Consistent with the findings of Hong et al. [3], the total phenol and flavonoid content in the leaves was highly significantly positively correlated with the antioxidant activity. The total phenol content in the flowers was highly significantly positively correlated with antioxidant activity, and flavonoid content was only significantly positively correlated with FRAP. Thus, total phenols and flavonoids can be considered important functional components for the antioxidant activity in loquat leaves and flowers. In addition, no obvious correlation was observed between antioxidant components and activity among the loquat leaves and flowers, which may be due to different types and contents of phenolic substances in the different tissues of loquat [33]. Yin et al. [34] reported that different tomato varieties have different phenolic compositions, and the correlation between phenolic composition and DPPH is closely related. It is believed that 5′-methoxymatairesinoside and 6-hydroxykaempferol-7-O-glucoside are the main components that contribute to differences in DPPH levels. Contrary to the present study’s findings, Zhou et al. [6] reported that the total phenol and flavonoid content in loquat flowers was highly significantly positively correlated with the three antioxidant activities. This difference may be due to the different materials used. A similar finding was observed in blackberry–raspberry hybrids and their parents, indicating that the correlation between antioxidant capacity and bioactive substances is dependent on the plant cultivar [35].

Comprehensive evaluation using principal component analysis can simplify multiple traits into several comprehensive variables while retaining original trait information and overcoming interference caused by the correlation between various traits [36]. Cluster analysis can group data with similar characteristics into one category according to its own internal guidelines to reduce errors caused by subjective judgment and to conduct comprehensive evaluation through data differences between various categories [37]. The comprehensive evaluation of plants based on principal component and cluster analyses has great application prospects in plant quality grading [38], trait evaluation [39,40], and the selection of superior hybrids [13,41]. In this study, the total phenolic content, flavonoid content, DPPH, ABTS, and FRAP of the F_1_ leaves and flowers of “Ninghaibai” and “Oobusa” were summarized into two principal components through principal component analysis, which can more objectively reflect the comprehensive antioxidant activity of loquat leaves and flowers. Ten strains with excellent quality were selected based on the comprehensive scores. The top three strains were ND107, “Oobusa”, and ND128. These strains had better comprehensive antioxidant activity and can be used for the future breeding of new loquat varieties. In addition, a total of 58 plants of “Ninghaibai” and “Oobusa” and their hybrid offspring were clustered into three categories through cluster analysis. The findings of the cluster analysis were consistent with the ranking of PCA analysis scores, and the top three strains with high antioxidant capacity—ND107, “Oobusa”, and ND128— were screened out. This indicates that it is feasible and reliable to use cluster and principal component analyses to comprehensively evaluate the antioxidant activity of loquat. In the future, these strains with high antioxidant activity in leaves and flowers could be utilized for the breeding of new loquat varieties. Additionally, their leaves and flowers hold significant value in producing functional beverages [42,43] and serving as natural food additives [44].

## 5. Conclusions

The genetic variation analysis of the five traits of the leaf and flower antioxidant components and activities of the F_1_-generation loquats “Ninghaibai” and “Oobusa” showed that hybrid breeding guided by the genetic characteristics of each trait can improve the possibility of cultivating new varieties with high antioxidant activity. The hybrid offsprings with strong antioxidant activity can be screened based on principal component and cluster analyses.

## Figures and Tables

**Table 1:** Genetic variation analysis of antioxidant components and activity in F_1_-generation leaves of loquats “Ninghaibai” and “Oobusa”.

Characteristic	Oobusa	Ninghaibai	Mid-Parent Value	F_1_	Separation Range	*CV*/%	*T*a/%	*MPH*/%	*HH*/%	*LH*/%	Kurt	Skew
Total phenols (mg GAE·g^−1^ FW)	242.98	169.38	206.18	165.05	84.74~296.34	21.22	80.05	−19.95	3.57	55.36	3.53	0.89
Flavonoids (mg RE·g^−1^ FW)	10.78	5.02	7.90	4.33	0.06~11.19	41.85	54.80	−45.20	1.79	69.64	3.53	0.64
DPPH (µmol TE·g^−1^ FW)	213.37	149.21	181.29	142.61	69.39~253.44	21.33	78.66	−21.34	1.79	59.93	2.72	0.58
ABTS (µmol TE·g^−1^ FW)	394.87	205.80	300.33	250.48	113.99~478.80	24.49	83.40	−16.60	3.57	19.64	3.03	0.95
FRAP (µmol ISE·g^−1^ FW)	828.24	560.88	694.56	480.01	146.61~814.53	23.50	69.11	−30.89	0	83.93	2.70	−0.61

**Table 2:** Genetic variation analysis of antioxidant components and activity in F_1_-generation flowers of loquats “Ninghaibai” and “Oobusa”.

Characteristic	Oobusa	Ninghaibai	Mid Parent Value	F_1_	Separation Range	*CV*/%	*T*a/%	*MPH*/%	*HH*/%	*LH*/%	Kurt	Skew
Total phenols (mg GAE·g^−1^ FW)	224.14	225.06	224.60	225.60	185.70~269.63	8.53	100.45	0.45	48.21	51.79	−0.32	0.10
Flavonoids (mg RE·g^−1^ FW)	8.72	8.31	8.51	8.18	5.70~10.96	13.81	96.13	−3.87	35.71	58.93	−0.25	0.96
DPPH (µmol TE·g^−1^ FW)	224.01	224.01	224.01	211.47	122.60~270.96	16.70	94.40	−5.60	41.07	58.93	−0.23	0.94
ABTS (µmol TE·g^−1^ FW)	293.06	306.70	299.88	317.24	209.74~429.41	14.14	105.79	5.79	51.79	28.57	0.08	0.11
FRAP (µmol ISE·g^−1^ FW)	638.25	664.69	651.47	597.27	460.98~769.48	10.95	91.68	−8.32	16.07	78.57	0.16	0.92

**Table 3:** The average value of antioxidant components and activity in flowers and leaves of three F_1_-generation groups of loquats “Ninghaibai” and “Oobusa”.

Characteristic		Population		
		I	II	III
Leaves	Total phenols (mg GAE·g^−1^ FW)	98.13	166.03	264.92
	Flavonoids (mg RE·g^−1^ FW)	0.82	4.40	10.21
	DPPH (µmol TE·g^−1^ FW)	88.41	143.60	223.81
	ABTS (µmol TE·g^−1^ FW)	158.91	249.20	427.59
	FRAP (µmol ISE·g^−1^ FW)	189.70	493.71	777.32
Flowers	Total phenols (mg GAE·g^−1^ FW)	236.30	225.43	213.54
	Flavonoids (mg RE·g^−1^ FW)	8.18	8.20	8.12
	DPPH (µmol TE·g^−1^ FW)	223.39	211.44	204.50
	ABTS (µmol TE·g^−1^ FW)	324.50	317.57	290.44
	FRAP (µmol ISE·g^−1^ FW)	612.78	598.23	596.46

**Table 4:** Factor loading, coefficient eigenvalues and variance contribution rates of principal components (PC).

	Characteristic	PC1	PC2
Leaves	Total phenols (mg GAE·g^−1^ FW)	0.934	0.177
	Flavonoids (mg RE·g^−1^ FW)	0.940	0.075
	DPPH (µmol TE·g^−1^ FW)	0.943	0.124
	ABTS (µmol TE·g^−1^ FW)	0.885	0.106
	FRAP (µmol ISE·g^−1^ FW)	0.923	0.168
Flowers	Total phenols (mg GAE·g^−1^ FW)	−0.266	0.883
	Flavonoids (mg RE·g^−1^ FW)	−0.120	0.537
	DPPH (µmol TE·g^−1^ FW)	−0.213	0.606
	ABTS (µmol TE·g^−1^ FW)	−0.210	0.599
	FRAP (µmol ISE·g^−1^ FW)	−0.055	0.868
Eigenvalues/%		4.458	2.640
Rate of variance/%		44.575	26.397
Rate of cumulative variances/%		44.575	70.973

**Table 5:** Comprehensive scores and rankings of antioxidant components and activity of three F_1_-generation taxa and their parent loquats “Ninghaibai” and “Oobusa”.

Germplasm No.	PC1		PC2		PC	
	Score	Rank	Score	Rank	Score	Rank
ND107	7.532	1	0.592	22	4.951	1
Oobusa	5.529	2	1.357	12	3.977	2
ND128	5.034	3	−0.842	43	2.849	3
ND082	1.369	8	1.674	9	1.482	4
ND167	1.028	12	2.235	4	1.477	5
ND142	0.205	25	3.474	2	1.421	6
ND036	1.614	6	0.794	18	1.309	7
ND135	2.365	5	−0.796	42	1.189	8
ND088	2.467	4	−1.216	45	1.097	9
ND093	0.626	20	1.832	7	1.075	10
ND120	0.646	19	1.495	10	0.962	11
ND121	0.688	16	1.313	13	0.920	12
ND139	1.414	7	−0.060	30	0.865	13
ND165	0.269	24	1.806	8	0.841	14
ND159	0.606	21	1.116	15	0.796	15
ND164	−0.972	43	3.459	3	0.676	16
ND123	−0.184	33	1.864	6	0.578	17
ND099	0.667	17	0.386	26	0.563	18
ND031	0.606	22	0.308	28	0.495	19
ND125	0.178	26	0.997	17	0.482	20
ND084	0.948	13	−0.355	35	0.463	21
ND050	−1.334	48	3.494	1	0.462	22
ND146	0.651	18	0.016	29	0.415	23
ND136	−0.270	35	1.466	11	0.376	24
Ninghaibai	0.158	27	0.656	21	0.343	25
ND156	0.540	23	−0.165	33	0.277	26
ND130	0.115	28	0.510	24	0.262	27
ND043	0.715	15	−0.750	41	0.170	28
ND124	−1.050	44	1.932	5	0.059	29
ND079	1.366	9	−2.192	53	0.043	30
ND116	1.083	10	−1.822	50	0.002	31
ND081	0.772	14	−1.371	46	−0.025	32
ND117	−0.147	32	−0.148	32	−0.147	33
ND090	−0.682	40	0.714	19	−0.163	34
ND162	0.069	31	−0.731	40	−0.229	35
ND069	1.037	11	−2.919	55	−0.434	36
ND045	−1.107	45	0.667	20	−0.447	37
ND166	−0.901	42	−0.175	34	−0.631	38
ND106	−1.292	47	0.407	25	−0.660	39
ND026	0.092	29	−1.963	52	−0.672	40
ND134	−0.532	37	−1.009	44	−0.709	41
ND154	−0.869	41	−0.643	37	−0.785	42
ND085	−0.359	36	−1.758	49	−0.880	43
ND086	−0.570	39	−1.461	47	−0.902	44
ND029	−1.886	53	0.556	23	−0.978	45
ND004	−0.557	38	−1.705	48	−0.984	46
ND122	−1.371	49	−0.721	39	−1.129	47
ND028	0.087	30	−3.246	58	−1.153	48
ND037	−1.860	52	−0.098	31	−1.205	49
ND119	−1.772	51	−0.358	36	−1.246	50
ND127	−0.263	34	−3.124	56	−1.327	51
ND089	−1.172	46	−1.941	51	−1.458	52
ND007	−2.704	55	0.342	27	−1.571	53
ND024	−1.496	50	−3.190	57	−2.126	54
ND080	−2.223	54	−2.238	54	−2.229	55
ND148	−4.359	56	1.206	14	−2.289	56
ND149	−5.629	58	1.020	16	−3.156	57
ND150	−4.911	57	−0.689	38	−3.341	58

## Data Availability

The raw data supporting the conclusions of this article will be made available by the authors on request.
